# Supplementary material for: Borylated 2,3,4,5-Tetrachlorophthalimide and Their 2,3,4,5-Tetrachlorobenzamide Analogues: Synthesis, Their Glycosidase Inhibition and Anticancer Properties in View to Boron Neutron Capture Therapy
Source: Molecules. 2022 May 26;27(11):3447. doi: 10.3390/molecules27113447 (PMC9182199; doi:10.3390/molecules27113447)
Supplement: Supplementary file 1 [file molecules-27-03447-s001.zip › molecules-1625530-Supplementary.pdf]

Supporting information

Borylated 2,3,4,5-tetrachlorophthalimide and their 2,3,4,5-tetrachlorobenzamide analogues: synthesis, their glycosidase inhibition and anticancer properties in view to Boron Neutron Capture Therapy

1. X-Ray crystallography data

1.1. Table S1. Crystal data and structure refinement for **meta 5**.

Empirical formula	$C_{19}H_{18}BCl_4NO_3$
Molecular mass	460.95 g/mol
Temperature	190(2) K
Wavelength	1.54184 Å
Crystal system	Triclinic
Space group	$P\bar{1}$
Unit cell dimensions	a = 9.7314(3) Å<br>α = 91.861(3)°<br>b = 13.4344(5) Å<br>β = 90.532(3)°<br>c = 17.4745(6) Å<br>γ = 111.062(3)°
Volume	2130.25(13) $Å^3$
Z	4
Density (calculated)	1.437 Mg/m^3
Absorption coefficient	5.221 mm^{-1}
F(000)	944
Crystal size	0.2 x 0.2 x 0.2 mm^3
Theta range for data collection	3.53 to 62.49°
Index ranges	-11<=h<=11, -15<=k<=15, -18<=l<=20
Reflections collected	18829
Independent reflections	6719 [R(int) = 0.0332]
Completeness to theta = 62.49°	98.8 %
Absorption correction	Semi-empirical from equivalents
Max. and min. transmission	1 and 0.63302
Refinement method	Full-matrix least-squares on F^2
Data / restraints / parameters	6719 / 0 / 505
Goodness-of-fit on F^2	1.147
Final R indices [I>2sigma(I)]	R1 = 0.0470, wR2 = 0.1418
R indices (all data)	R1 = 0.0555, wR2 = 0.1473
Largest diff. peak and hole	0.432 and -0.305 e.$Å^{-3}$

Table S2. Bond lengths [Å] and angles [°] for **meta 5**.

C(1A)-C(6A)	1.393(5)
C(1A)-C(2A)	1.398(5)
C(1A)-B(1A)	1.558(6)
C(2A)-C(3A)	1.385(5)
C(3A)-C(4A)	1.390(5)
C(3A)-N(1A)	1.425(5)
C(4A)-C(5A)	1.385(6)
C(5A)-C(6A)	1.393(6)
C(7A)-O(1A)	1.224(4)
C(7A)-N(1A)	1.344(5)
C(7A)-C(8A)	1.514(5)
C(8A)-C(13A)	1.381(5)
C(8A)-C(9A)	1.382(5)
C(9A)-C(10A)	1.393(5)
C(9A)-Cl(1A)	1.727(4)
C(10A)-C(11A)	1.393(6)
C(10A)-Cl(2A)	1.717(4)
C(11A)-C(12A)	1.380(5)
C(11A)-Cl(3A)	1.725(4)
C(12A)-C(13A)	1.381(5)
C(12A)-Cl(4A)	1.726(4)
C(14A)-O(2A)	1.464(5)
C(14A)-C(16A)	1.524(8)
C(14A)-C(17A)	1.530(7)
C(14A)-C(15A)	1.550(6)
C(15A)-O(3A)	1.472(5)
C(15A)-C(18A)	1.509(6)
C(15A)-C(19A)	1.518(6)
O(2A)-B(1A)	1.364(5)
O(3A)-B(1A)	1.363(5)
C(1B)-C(2B)	1.395(5)
C(1B)-C(6B)	1.403(5)
C(1B)-B(1B)	1.557(6)
C(2B)-C(3B)	1.379(5)
C(3B)-C(4B)	1.391(5)
C(3B)-N(1B)	1.423(5)
C(4B)-C(5B)	1.382(6)
C(5B)-C(6B)	1.378(6)
C(7B)-O(1B)	1.220(5)
C(7B)-N(1B)	1.351(5)
C(7B)-C(8B)	1.511(5)
C(8B)-C(9B)	1.381(5)
C(8B)-C(13B)	1.389(5)
C(9B)-C(10B)	1.391(5)
C(9B)-Cl(1B)	1.726(4)
C(10B)-C(11B)	1.394(5)
C(10B)-Cl(2B)	1.726(4)
C(11B)-C(12B)	1.384(5)
C(11B)-Cl(3B)	1.722(3)
C(12B)-C(13B)	1.381(5)
C(12B)-Cl(4B)	1.724(4)
C(14B)-O(2B)	1.467(5)
C(14B)-C(17B)	1.519(7)
C(14B)-C(16B)	1.523(6)
C(14B)-C(15B)	1.568(6)
C(15B)-O(3B)	1.462(5)
C(15B)-C(18B)	1.511(6)
C(15B)-C(19B)	1.520(6)
O(2B)-B(1B)	1.365(5)
O(3B)-B(1B)	1.357(5)
C(6A)-C(1A)-C(2A)	118.5(3)
C(6A)-C(1A)-B(1A)	122.1(3)
C(2A)-C(1A)-B(1A)	119.3(3)
C(3A)-C(2A)-C(1A)	121.1(3)

C(2A)-C(3A)-C(4A)	119.6(3)
C(2A)-C(3A)-N(1A)	121.9(3)
C(4A)-C(3A)-N(1A)	118.5(3)
C(5A)-C(4A)-C(3A)	120.0(3)
C(4A)-C(5A)-C(6A)	120.1(4)
C(5A)-C(6A)-C(1A)	120.5(3)
O(1A)-C(7A)-N(1A)	125.7(3)
O(1A)-C(7A)-C(8A)	120.1(3)
N(1A)-C(7A)-C(8A)	114.1(3)
C(13A)-C(8A)-C(9A)	119.8(3)
C(13A)-C(8A)-C(7A)	118.0(3)
C(9A)-C(8A)-C(7A)	122.1(3)
C(8A)-C(9A)-C(10A)	120.3(3)
C(8A)-C(9A)-Cl(1A)	119.4(3)
C(10A)-C(9A)-Cl(1A)	120.3(3)
C(9A)-C(10A)-C(11A)	119.6(3)
C(9A)-C(10A)-Cl(2A)	120.1(3)
C(11A)-C(10A)-Cl(2A)	120.3(3)
C(12A)-C(11A)-C(10A)	119.4(3)
C(12A)-C(11A)-Cl(3A)	120.4(3)
C(10A)-C(11A)-Cl(3A)	120.2(3)
C(11A)-C(12A)-C(13A)	120.8(3)
C(11A)-C(12A)-Cl(4A)	120.7(3)
C(13A)-C(12A)-Cl(4A)	118.6(3)
C(8A)-C(13A)-C(12A)	120.1(3)
O(2A)-C(14A)-C(16A)	106.7(4)
O(2A)-C(14A)-C(17A)	107.7(4)
C(16A)-C(14A)-C(17A)	112.2(4)
O(2A)-C(14A)-C(15A)	102.7(3)
C(16A)-C(14A)-C(15A)	113.2(4)
C(17A)-C(14A)-C(15A)	113.5(4)
O(3A)-C(15A)-C(18A)	108.2(3)
O(3A)-C(15A)-C(19A)	106.7(3)
C(18A)-C(15A)-C(19A)	109.8(4)
O(3A)-C(15A)-C(14A)	102.3(3)
C(18A)-C(15A)-C(14A)	115.7(4)
C(19A)-C(15A)-C(14A)	113.4(4)
C(7A)-N(1A)-C(3A)	123.9(3)
B(1A)-O(2A)-C(14A)	106.9(3)
B(1A)-O(3A)-C(15A)	106.8(3)
O(3A)-B(1A)-O(2A)	113.7(3)
O(3A)-B(1A)-C(1A)	124.0(3)
O(2A)-B(1A)-C(1A)	122.2(3)
C(2B)-C(1B)-C(6B)	118.1(3)
C(2B)-C(1B)-B(1B)	120.2(3)
C(6B)-C(1B)-B(1B)	121.5(3)
C(3B)-C(2B)-C(1B)	121.0(3)
C(2B)-C(3B)-C(4B)	120.0(3)
C(2B)-C(3B)-N(1B)	122.5(3)
C(4B)-C(3B)-N(1B)	117.5(3)
C(5B)-C(4B)-C(3B)	119.7(3)
C(6B)-C(5B)-C(4B)	120.3(4)
C(5B)-C(6B)-C(1B)	120.7(3)
O(1B)-C(7B)-N(1B)	125.6(3)
O(1B)-C(7B)-C(8B)	120.8(3)
N(1B)-C(7B)-C(8B)	113.5(3)
C(9B)-C(8B)-C(13B)	119.3(3)
C(9B)-C(8B)-C(7B)	122.7(3)
C(13B)-C(8B)-C(7B)	118.0(3)
C(8B)-C(9B)-C(10B)	120.2(3)
C(8B)-C(9B)-Cl(1B)	119.8(3)
C(10B)-C(9B)-Cl(1B)	119.9(3)
C(9B)-C(10B)-C(11B)	120.1(3)
C(9B)-C(10B)-Cl(2B)	120.3(3)
C(11B)-C(10B)-Cl(2B)	119.6(3)
C(12B)-C(11B)-C(10B)	119.3(3)
C(12B)-C(11B)-Cl(3B)	120.3(3)
C(10B)-C(11B)-Cl(3B)	120.4(3)
C(13B)-C(12B)-C(11B)	120.2(3)
C(13B)-C(12B)-Cl(4B)	119.0(3)
C(11B)-C(12B)-Cl(4B)	120.7(3)
C(12B)-C(13B)-C(8B)	120.7(4)
O(2B)-C(14B)-C(17B)	106.5(3)
O(2B)-C(14B)-C(16B)	107.7(3)
C(17B)-C(14B)-C(16B)	111.3(4)
O(2B)-C(14B)-C(15B)	101.9(3)
C(17B)-C(14B)-C(15B)	113.7(4)
C(16B)-C(14B)-C(15B)	114.8(4)
O(3B)-C(15B)-C(18B)	106.5(3)
O(3B)-C(15B)-C(19B)	108.3(3)
C(18B)-C(15B)-C(19B)	110.3(3)
O(3B)-C(15B)-C(14B)	102.0(3)
C(18B)-C(15B)-C(14B)	114.3(3)
C(19B)-C(15B)-C(14B)	114.6(4)
C(7B)-N(1B)-C(3B)	126.2(3)
B(1B)-O(2B)-C(14B)	106.9(3)
B(1B)-O(3B)-C(15B)	107.4(3)
O(3B)-B(1B)-O(2B)	113.7(3)
O(3B)-B(1B)-C(1B)	124.0(3)
O(2B)-B(1B)-C(1B)	122.3(3)

1.2. Table S3. Crystal data and structure refinement for **ortho 8**.

Empirical formula	$C_{20}H_{20}BCl_4NO_3$
Molecular mass	474.98
Temperature	190(2) K
Wavelength	0.71073 Å
Crystal system	Monoclinic
Space group	$P2_1/c$
Unit cell dimensions	a = 12.4312(9) Å<br>α = 90°<br>b = 18.6894(17) Å<br>β = 93.369(6)°.<br>c = 9.2372(7) Å<br>γ = 90°
Volume	2142.4(3) $Å^3$
Z	4
Density (calculated)	1.473 Mg/m^3
Absorption coefficient	0.575 mm^{-1}
F(000)	976
Crystal size	0.3 x 0.2 x 0.1 mm^3
Theta range for data collection	3.28 to 25.00°
Index ranges	-14<=h<=14, -12<=k<=22, -10<=l<=10
Reflections collected	8344
Independent reflections	8344 [R(int) = 0.0000]
Completeness to theta = 25.00°	99.8%
Absorption correction	Semi-empirical from equivalents
Max. and min. transmission	1 and 0.9793
Refinement method	Full-matrix least-squares on F^2
Data / restraints / parameters	8344 / 0 / 267
Goodness-of-fit on F^2	0.745
Final R indices [I>2sigma(I)]	R1 = 0.0611, wR2 = 0.1102
R indices (all data)	R1 = 0.1694, wR2 = 0.1272
Largest diff. peak and hole	0.498 and -0.309 e.$Å^{-3}$

Table S4. Bond lengths [Å] and angles [°] for **ortho 8**.

C(1)-C(6)	1.373(5)
C(1)-C(2)	1.393(5)
C(1)-C(7)	1.518(5)
C(2)-C(3)	1.389(5)
C(2)-Cl(1)	1.716(4)
C(3)-C(4)	1.389(5)
C(3)-Cl(2)	1.702(4)
C(4)-C(5)	1.400(5)
C(4)-Cl(3)	1.708(4)
C(5)-C(6)	1.388(5)
C(5)-Cl(4)	1.723(4)
C(7)-O(1)	1.222(4)
C(7)-N(1)	1.314(5)
C(8)-N(1)	1.460(4)
C(8)-C(9)	1.509(4)
C(9)-C(14)	1.389(5)
C(9)-C(10)	1.398(5)
C(10)-C(11)	1.392(4)
C(10)-B(1)	1.561(6)
C(11)-C(12)	1.376(5)
C(12)-C(13)	1.357(5)
C(13)-C(14)	1.369(5)
C(15)-O(2)	1.473(4)
C(15)-C(18)	1.496(6)
C(15)-C(17)	1.524(7)
C(15)-C(16)	1.531(5)
C(16)-O(3)	1.458(4)
C(16)-C(19)	1.493(5)
C(16)-C(20)	1.516(6)
B(1)-O(2)	1.345(5)
B(1)-O(3)	1.375(5)
C(6)-C(1)-C(2)	119.3(4)
C(6)-C(1)-C(7)	120.2(3)
C(2)-C(1)-C(7)	120.4(4)
C(3)-C(2)-C(1)	121.2(4)
C(3)-C(2)-Cl(1)	119.9(3)
C(1)-C(2)-Cl(1)	118.9(3)
C(4)-C(3)-C(2)	119.4(3)
C(4)-C(3)-Cl(2)	120.3(3)
C(2)-C(3)-Cl(2)	120.3(3)
C(3)-C(4)-C(5)	119.1(3)
C(3)-C(4)-Cl(3)	120.4(3)
C(5)-C(4)-Cl(3)	120.5(3)
C(6)-C(5)-C(4)	120.7(4)
C(6)-C(5)-Cl(4)	119.4(3)
C(4)-C(5)-Cl(4)	119.8(3)
C(1)-C(6)-C(5)	120.2(4)
O(1)-C(7)-N(1)	125.3(4)
O(1)-C(7)-C(1)	118.7(4)
N(1)-C(7)-C(1)	115.9(4)
N(1)-C(8)-C(9)	115.7(3)
C(14)-C(9)-C(10)	119.3(3)
C(14)-C(9)-C(8)	121.2(4)
C(10)-C(9)-C(8)	119.5(3)
C(11)-C(10)-C(9)	117.5(3)
C(11)-C(10)-B(1)	116.8(4)
C(9)-C(10)-B(1)	125.6(3)
C(12)-C(11)-C(10)	122.6(4)
C(13)-C(12)-C(11)	118.8(4)
C(12)-C(13)-C(14)	120.7(4)
C(13)-C(14)-C(9)	121.0(4)
O(2)-C(15)-C(18)	109.4(3)
O(2)-C(15)-C(17)	106.5(4)
C(18)-C(15)-C(17)	111.2(4)
O(2)-C(15)-C(16)	103.0(3)
C(18)-C(15)-C(16)	114.1(4)
C(17)-C(15)-C(16)	112.0(4)
O(3)-C(16)-C(19)	108.9(3)
O(3)-C(16)-C(20)	106.2(3)
C(19)-C(16)-C(20)	107.6(4)
O(3)-C(16)-C(15)	102.5(3)
C(19)-C(16)-C(15)	116.7(4)
C(20)-C(16)-C(15)	114.1(4)
O(2)-B(1)-O(3)	113.0(4)
O(2)-B(1)-C(10)	127.3(4)
O(3)-B(1)-C(10)	119.6(4)
C(7)-N(1)-C(8)	122.3(3)
B(1)-O(2)-C(15)	107.2(3)
B(1)-O(3)-C(16)	107.5(3)

2. NMR Spectra

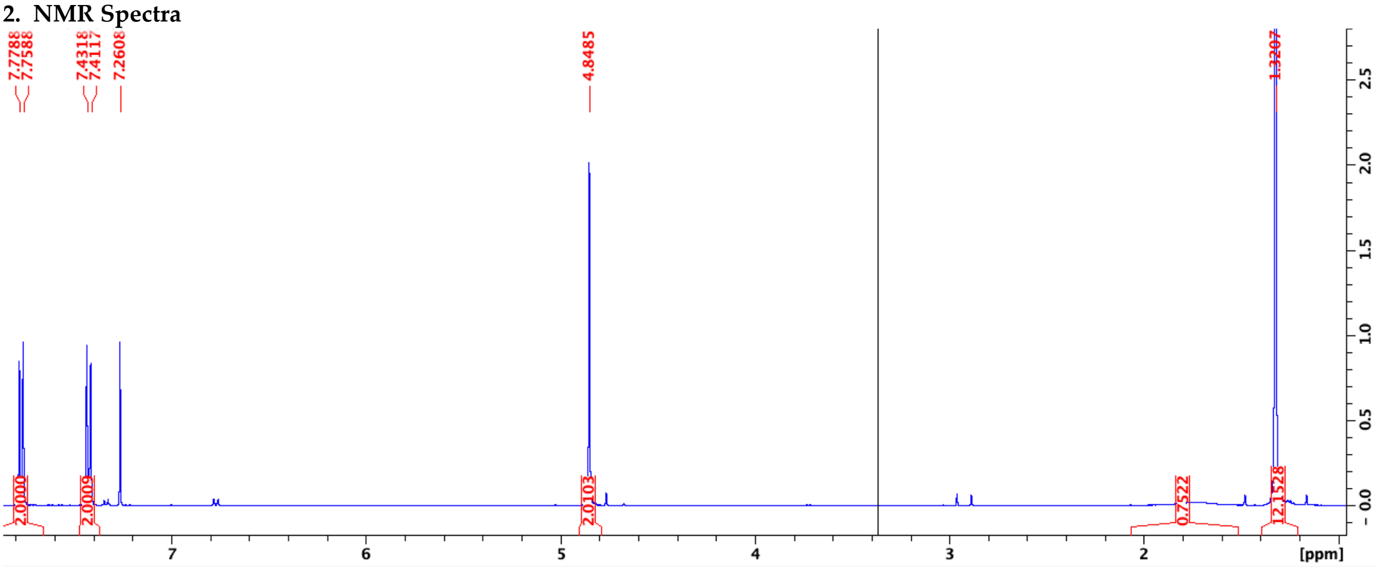

Figure S1. ^{1}H NMR spectrum of **para 3**.

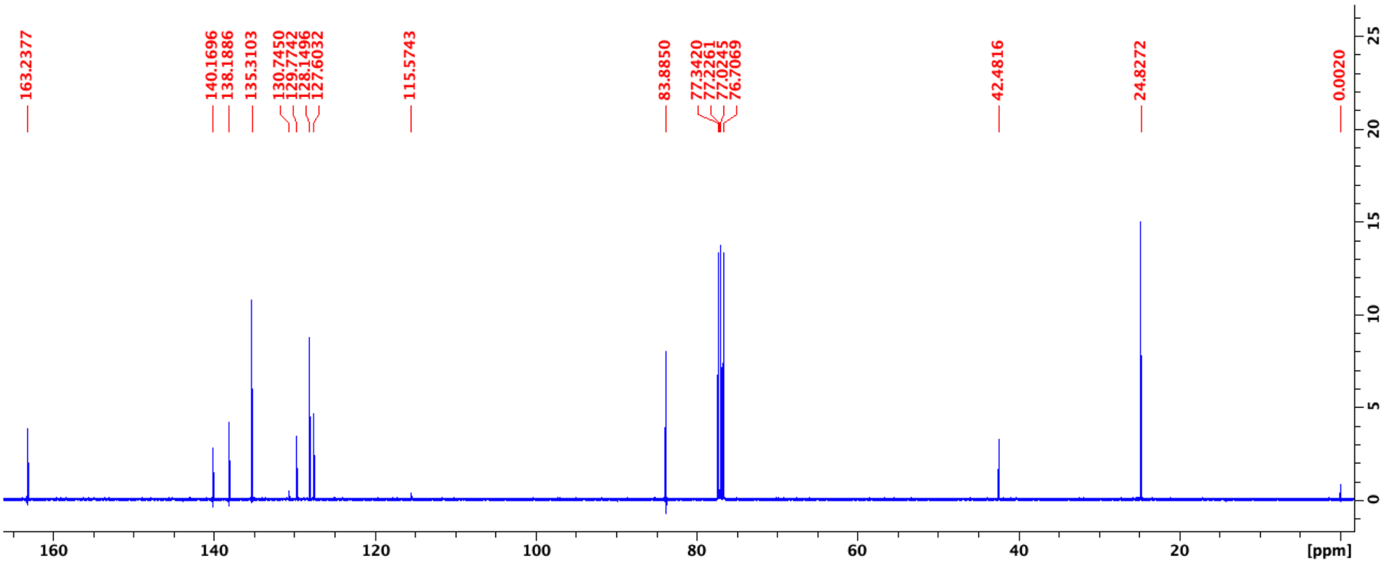

Figure S2. ^{13}C NMR spectrum of **para 3**.

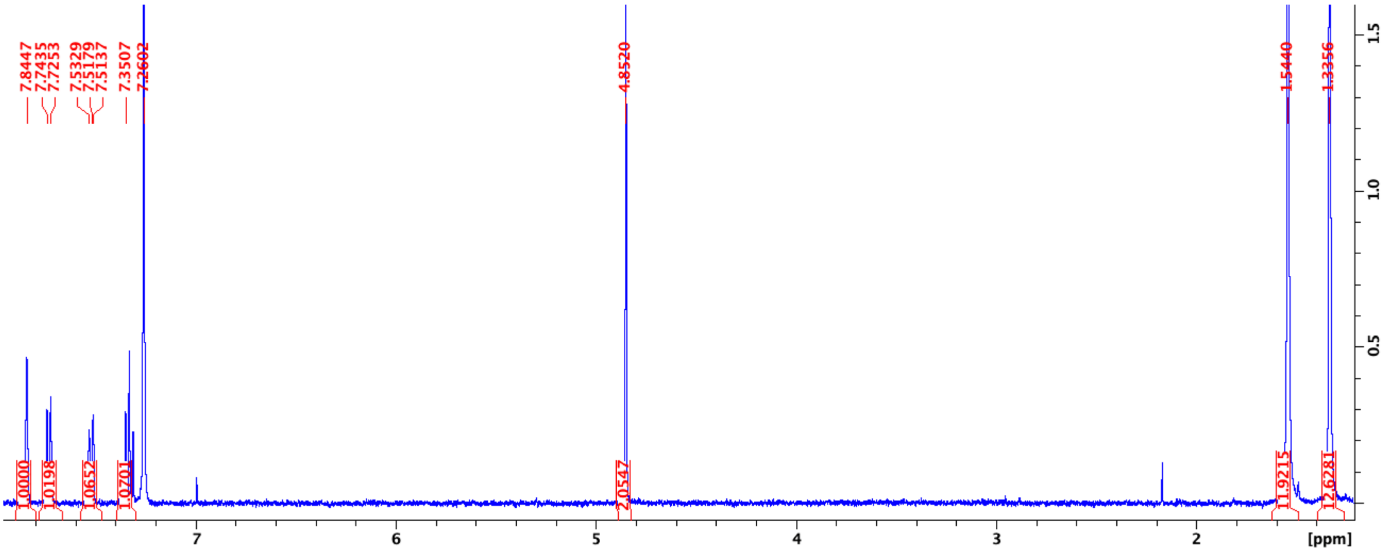

Figure S3. ^{1}H NMR spectrum of **meta 3**.

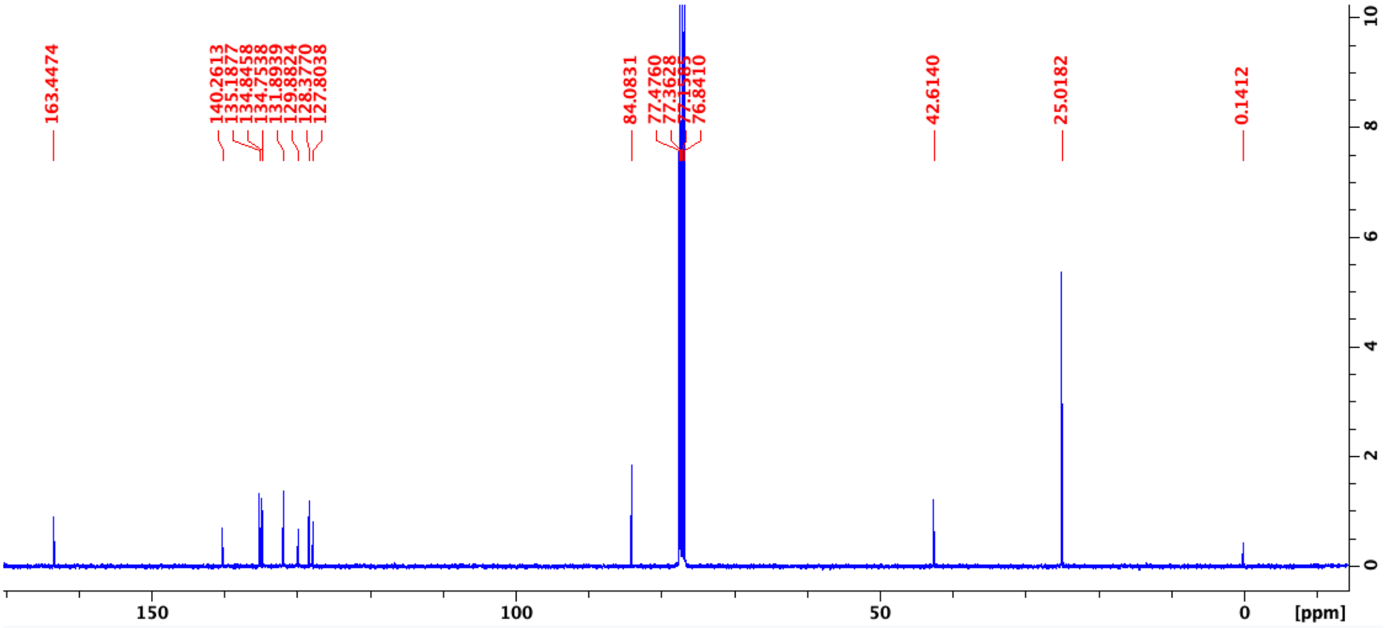

Figure S4. ^{13}C NMR spectrum of **meta 3**.

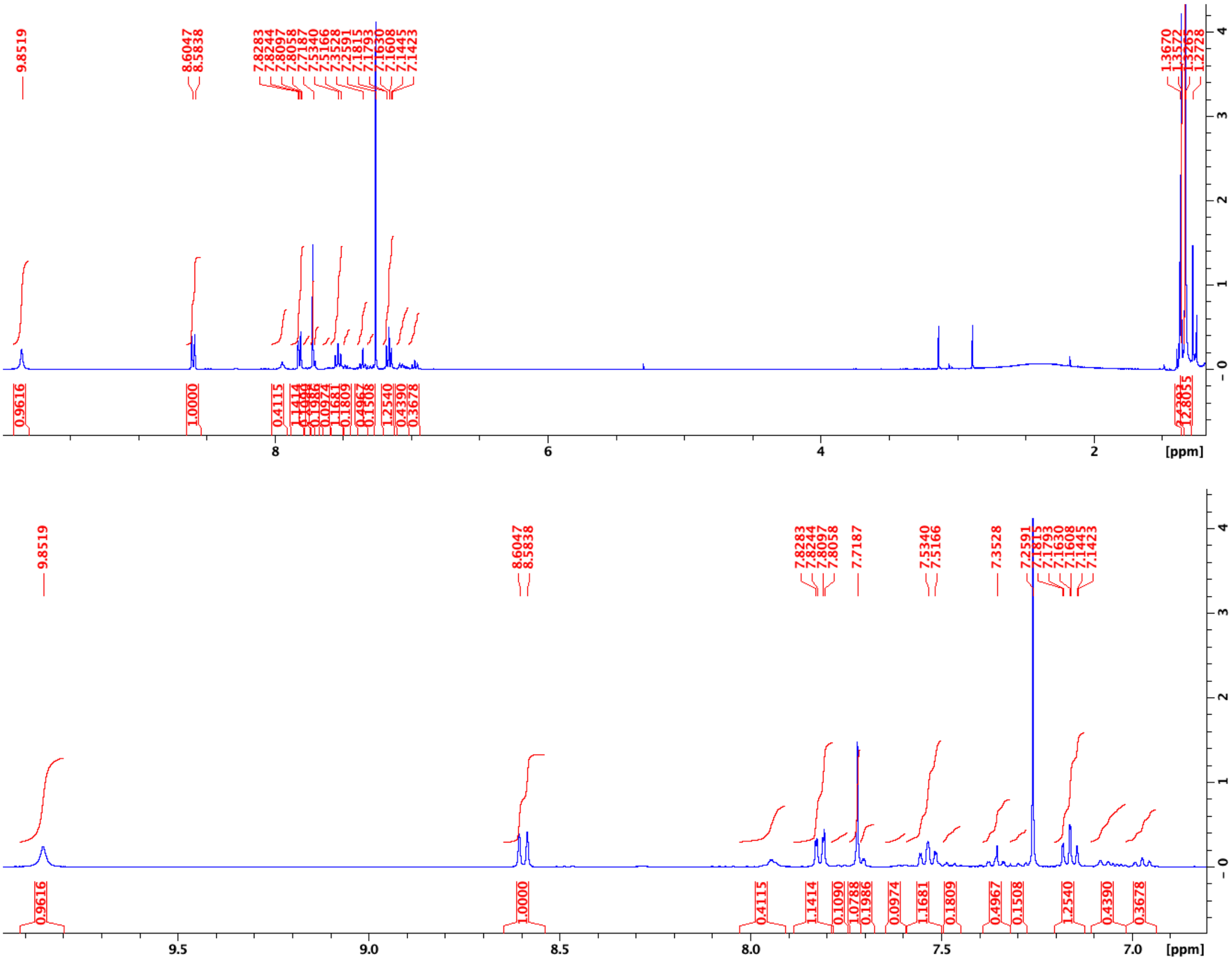

Figure S5. ^{1}H NMR spectrum of **ortho 5**.

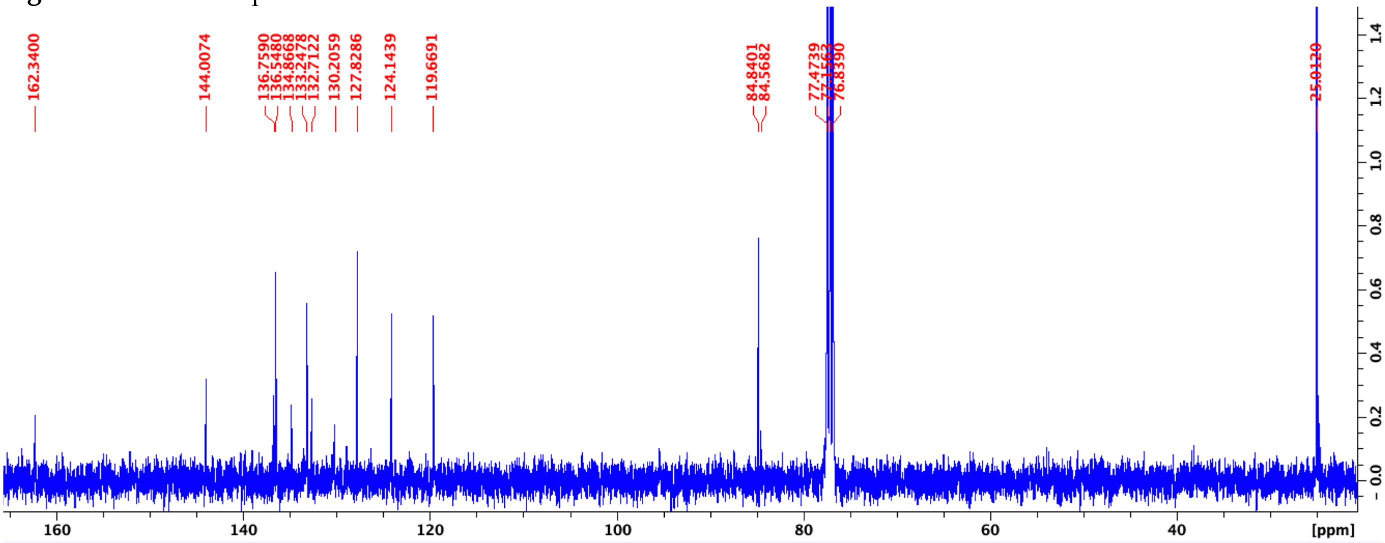

Figure S6. ^{13}C NMR spectrum of **ortho 5**.

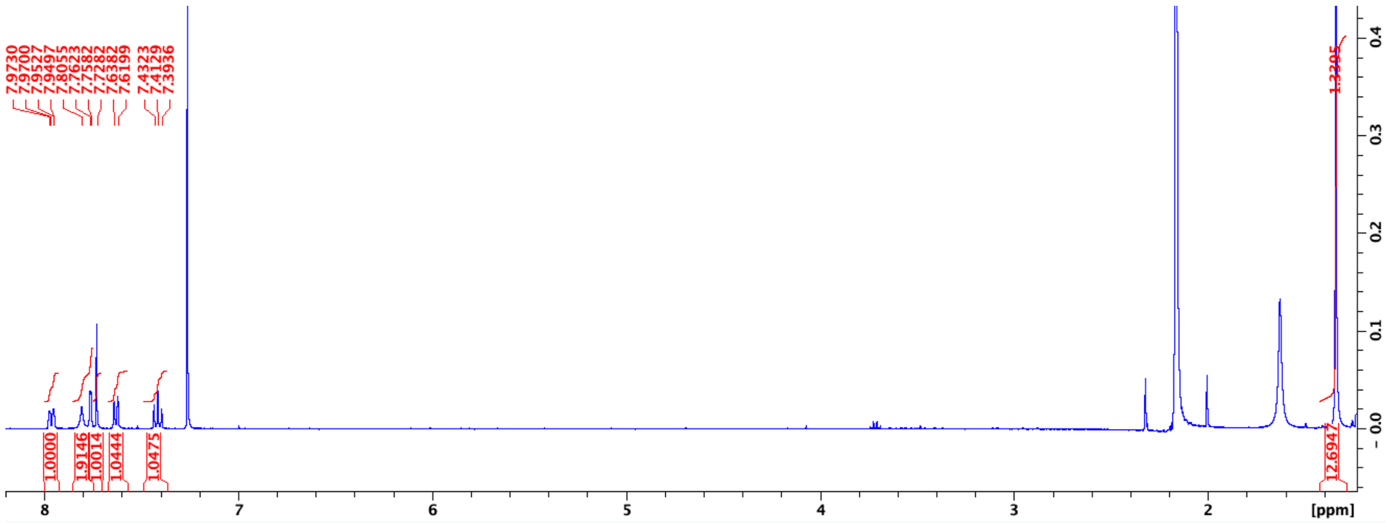

Figure S7. ^{1}H NMR spectrum of **meta 5**.

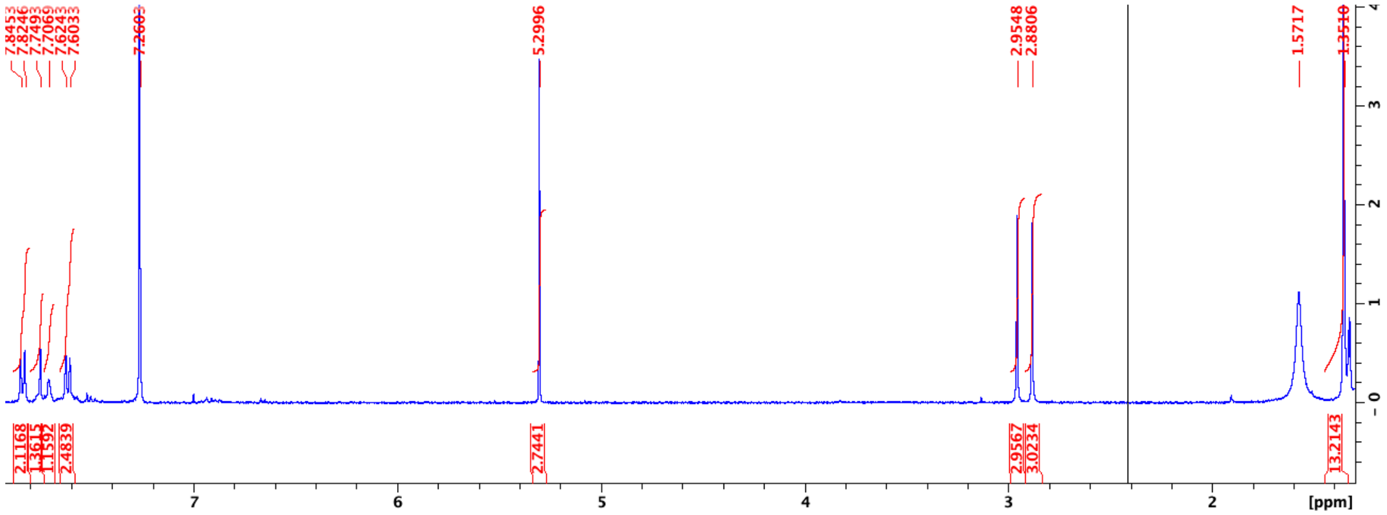

Figure S8. ^{1}H NMR spectrum of **para 5**.

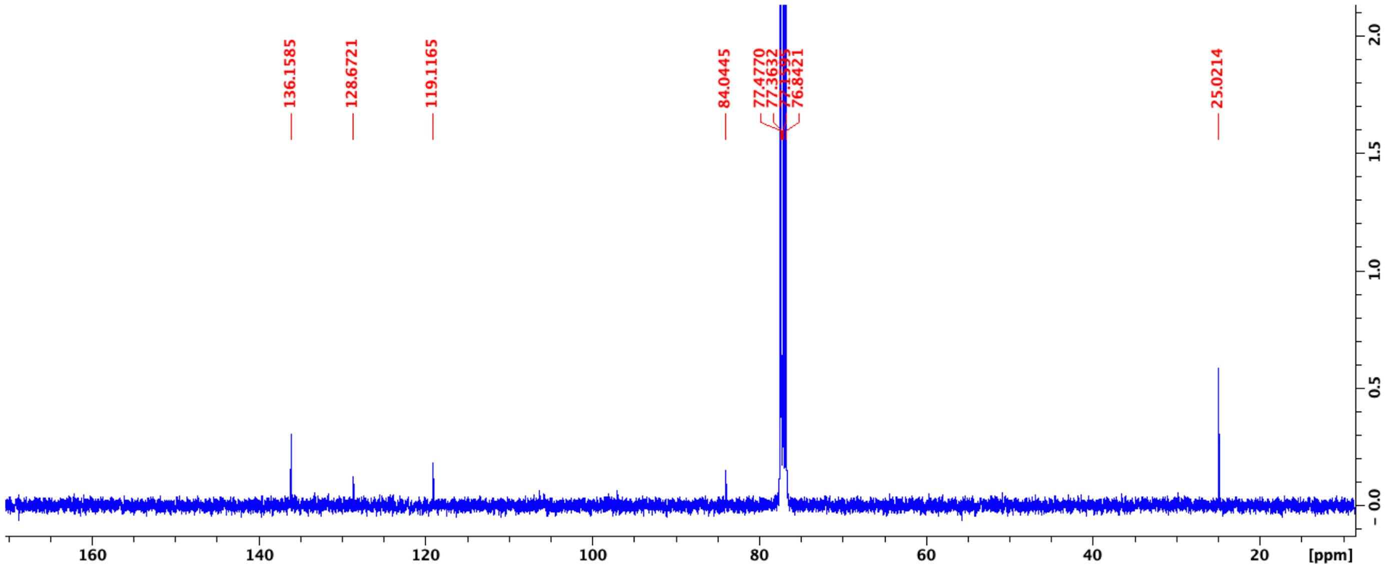

Figure S9. ^{13}C NMR spectrum of **para 5**.

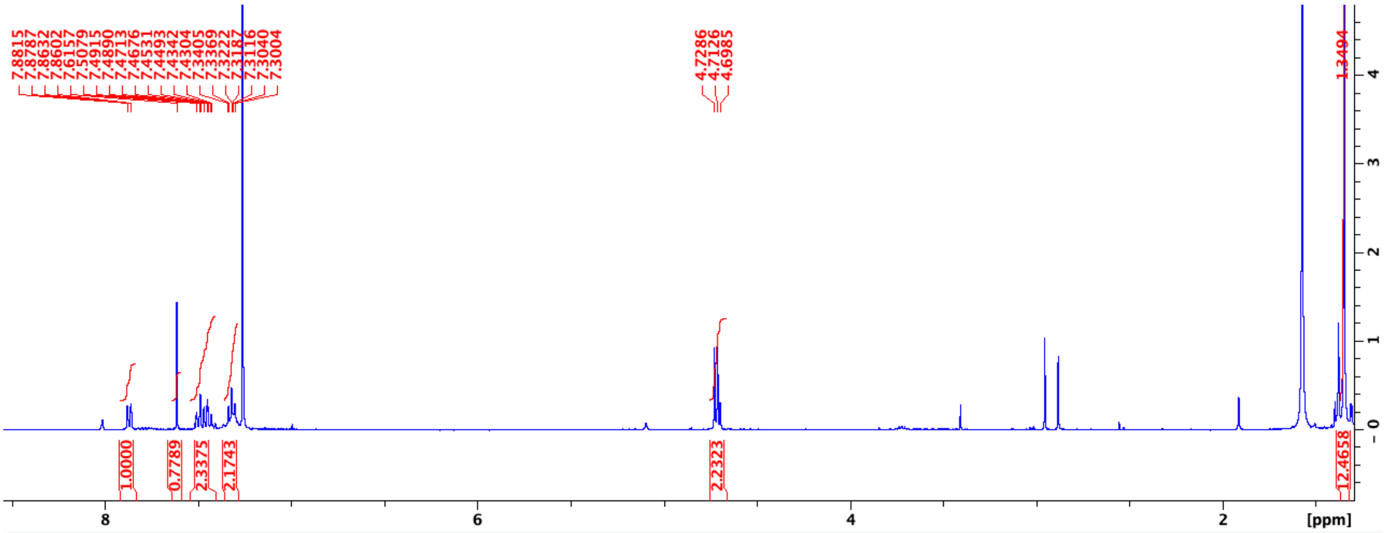

Figure S10. ^{1}H NMR spectrum of **ortho 8**.

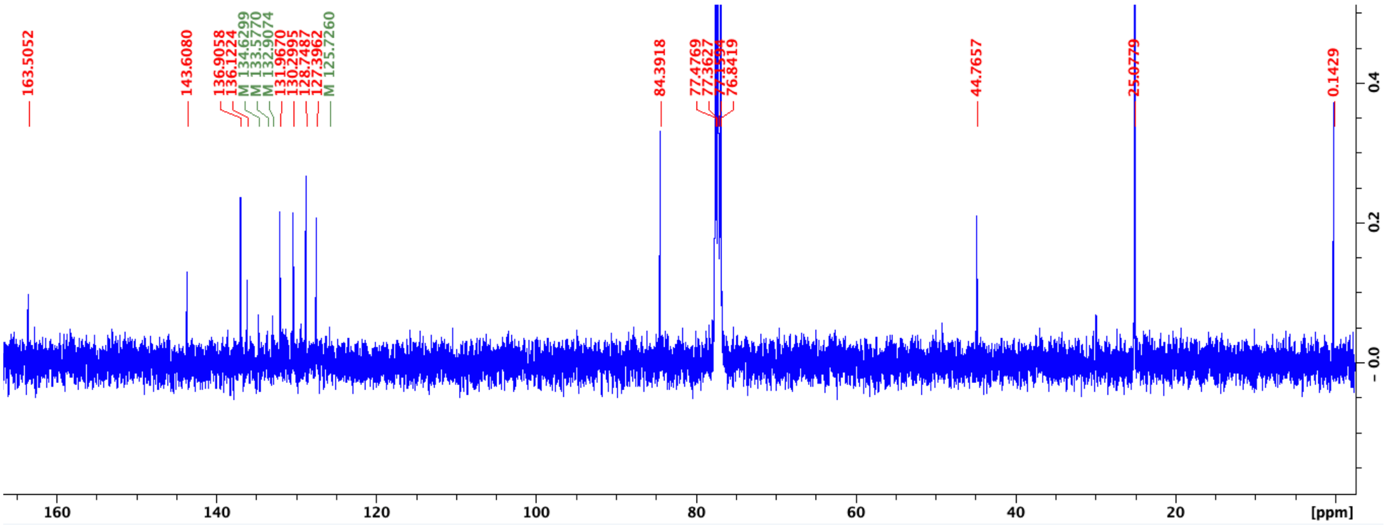

Figure S11. ^{13}C NMR spectrum of **ortho 8**.

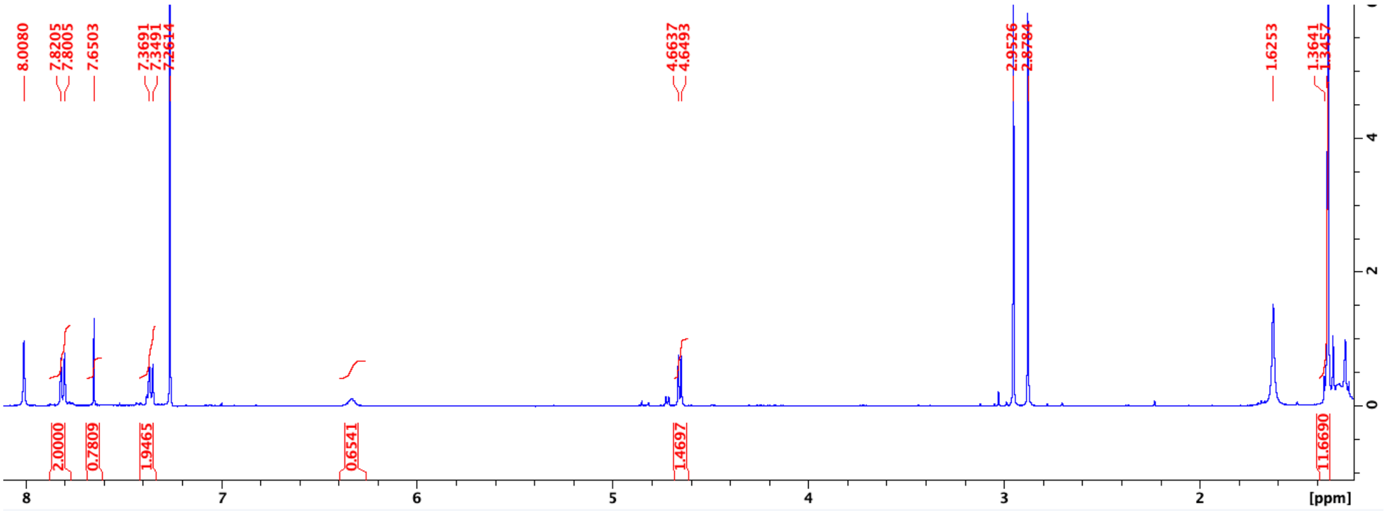

Figure S12. ^{1}H NMR spectrum of **para 8**.

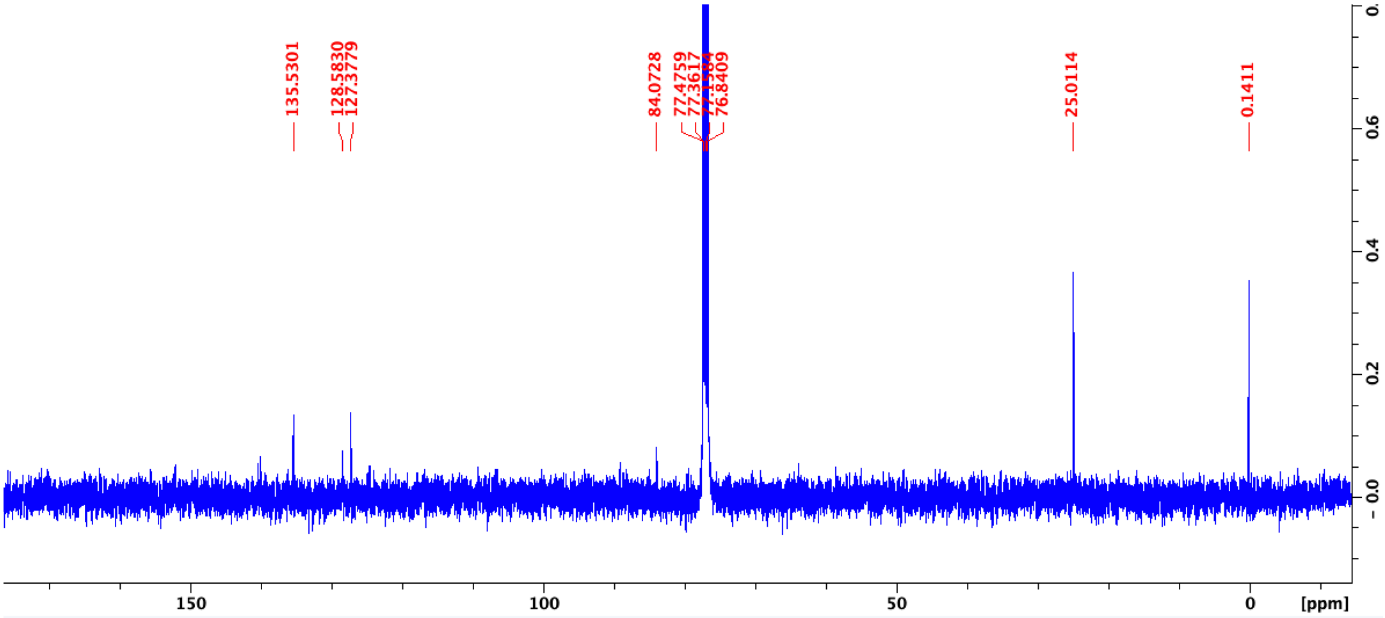

Figure S13. ^{13}C NMR spectrum of **para 8**.
